# Molecular Mechanisms of Microbial Survivability in Outer Space: A Systems Biology Approach

**DOI:** 10.3389/fmicb.2020.00923

**Published:** 2020-05-15

**Authors:** Tetyana Milojevic, Wolfram Weckwerth

**Affiliations:** ^1^Extremophiles/Space Biochemistry Group, Department of Biophysical Chemistry, University of Vienna, Vienna, Austria; ^2^Department of Ecogenomics and Systems Biology, University of Vienna, Vienna, Austria; ^3^Vienna Metabolomics Center, University of Vienna, Vienna, Austria

**Keywords:** microbes in space, space missions, –omics technology, extremophiles, outer space

## Abstract

Since the dawn of space exploration, the survivability of terrestrial life in outer space conditions has attracted enormous attention. Space technology has enabled the development of advanced space exposure facilities to investigate *in situ* responses of microbial life to the stress conditions of space during interplanetary transfer. Significant progress has been made toward the understanding of the effects of space environmental factors, e.g., microgravity, vacuum and radiation, on microorganisms exposed to real and simulated space conditions. Of extreme importance is not only knowledge of survival potential of space-exposed microorganisms, but also the determination of mechanisms of survival and adaptation of predominant species to the extreme space environment, i.e., revealing the molecular machinery, which elicit microbial survivability and adaptation. Advanced technologies in –omics research have permitted genome-scale studies of molecular alterations of space-exposed microorganisms. A variety of reports show that microorganisms grown in the space environment exhibited global alterations in metabolic functions and gene expression at the transcriptional and translational levels. Proteomic, metabolomic and especially metabolic modeling approaches as essential instruments of space microbiology, synthetic biology and metabolic engineering are rather underrepresented. Here we summarized the molecular space-induced alterations of exposed microorganisms in terms of understanding the molecular mechanisms of microbial survival and adaptation to drastic outer space environment.

## Introduction

**FIGURE 1:**
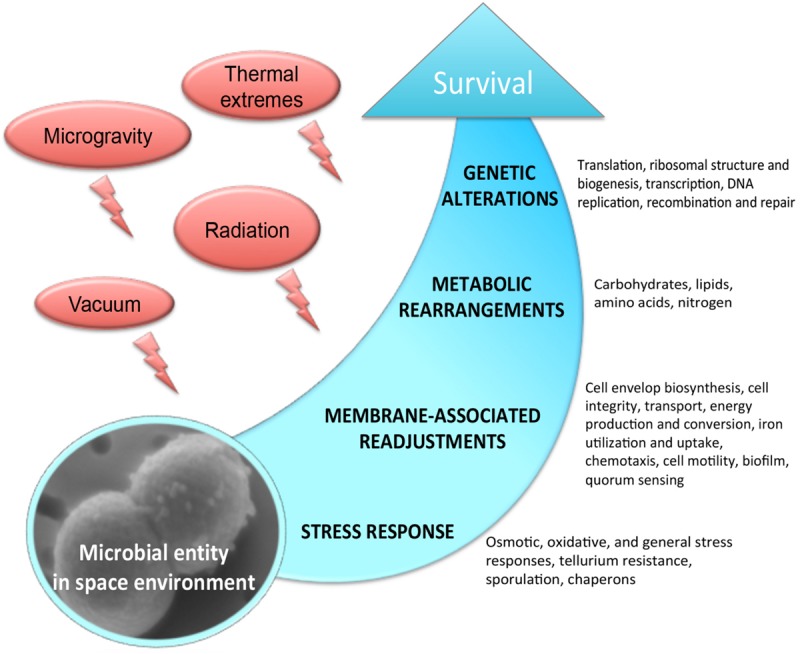
Molecular response experienced by microorganisms in the outer space environment.

The outer space environment, which is characterized by a high vacuum and an intense radiation, provides hostile conditions to any form of life. With the upcoming long-term space explorations, it is becoming increasingly important to understand the molecular mechanisms of survival in outer space. Remarkably, a few extremophilic microbial species have been shown to withstand the drastic influence of the outer space factors (Saffary et al., 2002; Sancho et al., 2007; Cockell et al., 2011; Wassmann et al., 2012; Kawaguchi et al., 2013; Selbmann et al., 2015), and numerous studies have significantly proved the possibility of microbial life transfer through space (Horneck et al., 2008; Nicholson, 2009). Considerable progress has been made toward the understanding of the effects of space environmental factors, e.g., microgravity, vacuum and radiation, on microorganisms exposed to real and simulated space conditions. However, we still have been missing an explicit knowledge of molecular mechanisms permitting survival and adaptation in the outer space environment. Space parameters affect microorganisms by altering a variety of physiological features, including cell metabolism, proliferation rate, cell division, cell motility, virulence, and biofilm production (Figure 1). These physiological perturbations of space-exposed microorganisms are very poorly understood at the molecular level. In this context, omics-based analyses combined with classical phenotyping and physiological measurements provide the integrative suite of tools to functionally decipher the mechanisms of microbial survivability in outer space. Exploring the relevant mechanisms underlying metabolic and physiological changes which microorganisms experience during exposure to outer space, the omics-based approach integrates the different fragments of biological information to understand the flow of information from genomes, mRNA, proteins to metabolites (Weckwerth, 2011a; Ott et al., 2017, 2019b,a), and explains how the microorganisms adapt to this extreme environment.

Systems biology is an interdisciplinary field that incorporates the results of genome-scale molecular analysis–omics techniques–such as genomics, transcriptomics, proteomics, and metabolomics and genome-scale metabolic and regulatory biomathematical models to reveal complex molecular interactions underlying molecular evolution, functional and phenotypical diversity and molecular adaptation (Kitano, 2000; Ideker et al., 2001; Weckwerth, 2003, 2011a,b). The roots of systems biology go back to the development of the General System Theory by Ludwig von Bertalanffy in the early 30’s of the 20th century. He published one of the first explicit articles about open systems, feedback regulation and self-organization in living systems and proposed the basic mathematical framework for systems biology (Bertalanffy, 1940, 1969; Weckwerth, 2016, 2019).

In this review we focus on molecular mechanisms of microbial survivability in the outer space environment revealed with the help of global and integrative –omics approaches of systems biology that have been recently used to study microorganisms exposed to real and simulated space conditions. The use of –omics in space life sciences potentially has a pivotal role in the understanding of the molecular machineries implicated by microorganisms to tolerate the harsh conditions of space.

## Metabolic Changes

Exposure to space environment strongly modifies the expression of genes and the abundance of proteins related to metabolism (Table 1). Space parameters primarily affect the genes involved in metabolism, raising further alterations of the microbial growth. However, energy and regenerative power are needed for the microbial cell to counteract the drastic effects of space exposure by launching a number of regenerative activities and repair mechanisms.

**TABLE 1:** Omics studies of the microbial physiology in real and simulated outer space environment.

**Microorganism studied**	**Exposure conditions**	**Research platform**	**Physiological effect**	**Molecular alterations**	**References**
*Aspergillusfumigatus, Cladosporium cladosporioides*	UVC Simulated Martian Conditions	TMT (tandem mass tag) LC/MS Orbitrap Fusion Tribrid mass spectrometer	Differential abundance of proteins involved in ribosome biogenesis, translation, and carbohydrate metabolic processes was observed	Differential abundance of proteins involved in ribosome biogenesis, translation, and carbohydrate metabolic processes	Blachowicz et al., 2019
*Candida albicans*	Simulated microgravity	qRT-PCR	Increased filamentous forms, morphogenic switch consistent with enhanced pathogenicity	Gene expression changes related to budding, separation and yeast hyphal transition	Altenburg et al., 2008
*Candida albicans*	Cultivation aboard NASA Shuttle Atlantis STS-115	Microarray Agilent platform	Enhanced aggregation and random budding	Downregulation of ergosterol-encoding genes and genes involved in actin cytoskeleton; induction of ABC transporters and members of the major facilitator family; up-regulation of genes involved in oxidative stress resistance	Crabbé et al., 2013
*Cupriavidus metallidurans* strain CH34	Simulated microgravity	ICPL MudPIT MALDI-TOF-MS	A switch to anoxic or microoxic conditions	Differentially abundant universal stress proteins, cold shock proteins, nitrate reductase, and proteins involved in transport activity	Leroy et al., 2010
*Bacillus cereus* strains LCT-BC25 and LCT-BC235	398 h space flight (Tiangong-1 space station)	Illumina HiSeq 2000 sequencer 2D-LC-MS/MS ESI-MS/MS using the TripleTOF 5600 System	Significantly slower growth rate; significantly higher amikacin resistance level; changes in metabolism	Three polymorphic loci in the flight strains LCT-BC25 and LCT-BC235; differential expression of genes and abundance of proteins relevant to metabolism, structural function, gene expression modification and translation, and virulence	Su et al., 2014
*Bacillus subtilis* spores	559-day space mission (ISS) Simulated Martian conditions	Microarray Agilent platform	Broader and more severe stress response of spores exposed to space than spores exposed to simulated Martian conditions	Increased transcript levels of stress-related regulons responding to DNA damage (SOS response, SPbprophage induction), protein damage (CtsR/Clp system), oxidative stress (PerR regulon), and cell envelope stress (SigV regulon)	Nicholson et al., 2012
*Bacillus subtilis*	Spaceflight aboard the ISS (BRIC-21 and BRIC-23)	Illumina HiSeq 4000 platform	Differences in oxygen availability between flight and ground control samples, likely due to differences in cell sedimentation and the toroidal shape assumed by the liquid cultures in microgravity	Upregulated genes involved in biofilm formation, biotin and arginine biosynthesis, siderophores, manganese transport, toxin production and resistance, and sporulation inhibition	Morrison et al., 2019
*Bacillus pumilus*	18-month space mission (ISS) Simulated space conditions	Fluorescence two-dimensional difference gel electrophoresis (2D-DiGE) and mass spectrometry based proteomic analysis	Enhanced UVC resistance of “space-surviving” strains (spores and vegetative cells)	Differentially abundant proteins involved in menaquinone biosynthesis, electron transport, ribosome structure, transcription, spore thermostability, and oxidative stress response	Vaishampayan et al., 2012
*Bacillus pumilus*	18 months on-board the ISS	LC-MS/MS Orbitrap Fusion Tribrid	Enhanced resistance to UV irradiation and oxidative stress	Increased abundance of proteins related to survival, growth advantage, and stress response	Chiang et al., 2019
*Escherichia coli*	Spaceflight aboard the ISS	RNA-Seqwith Illumina platform	Adapted to grow at higher antibiotic concentrations in space compared to Earth	Specific responses related to oxidative stress and starvation response	Aunins et al., 2018
*Escherichia coli* strain K12 MG1655	Simulated microgravity	Microarray Affymetrix Analysis RT-PCR	The enhanced growth in simulated gravity conditions; glycerol supplementation of cultivation medium reduced multiple stress responses to microgravity	Up-regulation of genes encoding adaptation to stress (*sufE* and *ssrA*) and involved in DNA replication (*srmB*); down-regulation of genes encoding membrane transporters (*ompC*, *exbB*, *actP*, *mgtA*, *cysW* and nikB), carbohydrate catabolic processes (*ldcC*, *ptsA*, *rhaD* and *rhaS*) and nucleoside metabolism (*dfp*, *pyrD* and *spoT*)	Arunasri et al., 2013
*Deinococcus radiodurans* R1	Simulated UVC and vacuum conditions	Shotgun proteomics with HPLC nESI-MS/MS using Orbitrap Elite Metabolomics analysis with LECO Pegasus^®^ 4D GC × GC-TOF spectrometer	Relative survival rate of 65% for UVC/vacuum exposed cells compared to control conditions; preserved cellular integrity, no detectable damage of cell surface and cell morphology	Differentially abundant proteins involved in TCA cycle, DNA damage response systems (PolA, PprA, GyrA/B, DdrB, DdrD, UvrB, recQ, ruvABC, MutT, MutS2, and Mrr restriction protein), ROS scavenging systems (pyridoxal 5′-phosphate synthase, peroxidase, sulfoxide reductase MsrA, thioredoxin reductase, PdxS and PdxT), and transcriptional regulators (DdrO and CRP regulon); up-regulation of protein functional categories of cysteine, methionine and tryptophan metabolism, RNA degradation, and aminoacyl-tRNA biosynthesis; significantly increased abundance of ethanolamine (cellular supply of reduced nitrogen and precursor for acetyl CoA), O-Palmytoyl-L-Carnitine chloride (quaternary amine and compatible solute which impacts bacterial survival in extreme conditions), and octadecanoic (stearic) acid (biofilm-associated compound)	Ott et al., 2017
*Deinococcus radiodurans* R1	Simulated vacuum conditions	Shotgun proteomics with HPLC nESI-MS/MS using Orbitrap Elite Metabolomics analysis with LECO Pegasus^®^ 4D GC × GC-TOF spectrometer	After 90 days of high vacuum exposure, survival of *D. radiodurans* cells was 2.5-fold lower compared to control cells	Proteases, tRNA ligases, reactive oxygen species (ROS) scavenging proteins, nucleic acid repair proteins, TCA cycle proteins, and *S*-layer proteins are highly abundant after vacuum exposure. The overall abundance of amino acids and TCA cycle intermediates is reduced during the recovery phase of *D. radiodurans* as they are needed as carbon source; upregulation of Type III histidine kinases	Ott et al., 2019a
*Deinococcus radiodurans* R1	Simulated microgravity	Shotgun proteomics with HPLC nESI-MS/MS using Orbitrap Elite Metabolomics analysis with LECO Pegasus^®^ 4D GC × GC-TOF spectrometer	Growth under simulated microgravity causes an increased demand for amino acids	Increased abundance of several proteins associated with processes involving DNA, such as DR_2410 (DnaX), DR_1707 (PolA), DNA ligase DR_2069 (LigA) and the transcription repair coupling factor DR_1532 (Mfd); increased abundance of cell envelope-associated proteins.	Ott et al., 2019b
*Klebsiella pneumonia* strain LCT-KP289	398 h space flight (Tiangong-1 space station)	Illumina HiSeq 2000 sequencer	A higher cotrimoxazole resistance level of flight strain	Differentially expressed genes and proteins involved in energy production and conversion, carbohydrate transport and metabolism, translation, ribosomal structure and biogenesis, posttranslational modification, protein turnover, and chaperone functions; synonymous mutation of the ytfG gene, which may influence fructose and mannose metabolic processes of flight strain.	Guo et al., 2015
*Klebsiella pneumoniae* strain ATCC BAA-2146	15-day space mission. Simulated space condition with microgravity	Illumina HiSeq 2000 sequencer RNA-Seq and comparative transcriptomics Quantitative RT-PCR	Strain-specific mutations, elongated forms, reduced hydrogen peroxide (H_2_O_2_) tolerance and increased biofilm formation ability of flight strain	Differentially expressed genes involved in amino acid transport and metabolism, and carbohydrate transport and metabolism; several differentially regulated non-coding RNAs (ncRNAs)	Li et al., 2014
*Klebsiella pneumoniae*	398 h space flight (Tiangong-1 station)	Illumina HiSeq 2000 sequencer	Increased strain diversity Acquired drug resistance	Large number of mutant genes related to transport and metabolism, including the gene encoding dihydroxyaceton kinase, which generates the ATP and NADH required for microbial growth	Guo et al., 2014
*Pseudomonas aeruginosa* PAO1	9-day spaceflight (ISS) Simulated microgravity	MudPIT via nano LC-MS/MS Microarray Affymetrix GeneChip analysis	Adaptation to an anaerobic mode of growth during spaceflight	Differentially expressed genes and differentially abundant proteins associated with growth under anaerobic conditions, virulence, nitrogen metabolism, purine and pyrimidine metabolism, fatty acid biosynthesis, oxidative phosphorylation, ribosome synthesis, and transcription with Hfq regulon as global transcriptional regulator involved	Crabbé et al., 2011
*Rhodospirillum rubrum* S1H	10 and 12-day space flights (ISS). Simulated microgravity and space-ionizing radiation.	Microarray platform High throughput gel-free proteomics with Isotope-Coded Protein Label (ICPL) technology Multi-Dimensional Protein Identification Technology (MudPIT)	Minimized effect of microgravity; increased sensitivity to ionizing radiation during space mission	Differential expression of genes and abundance of proteins associated with translation, transcription, ribosomal structure and biogenesis, energy production and conversion, putative oxidative and osmotic stress, tellurium resistance, solute transport and osmotic regulation	Mastroleo et al., 2009
*Rhodospirillum rubrum* S1H	Simulated microgravity	Microarray platform ICPL MudPIT LC-QqQLIT-MS liquid chromatography coupled to hybrid quadrupole-linear ion trap mass spectrometry	Higher pigmentation, no change in cell density and culture oxygenation	Elevated components of the N- acylhomoserine lactone (AHL)-type quorum sensing (QS)-system. Differentially expressed genes and differentially abundant proteins associated with membrane-bound photosynthetic apparatus, cell envelope biogenesis, ribosomal structure and biogenesis, transcription, lipid metabolism, amino acid and carbohydrate transport and metabolism, stress response, inorganic ion transport, chemotaxis	Mastroleo et al., 2013
*Saccharomyces cerevisiae*	12-day space flight (ISS)	2D-PAGE MALDI-TOF/TOF MS	Altered budding patterns; a switch toward more random budding	Increased protein degradation; ubiquitin presence in the microgravity samples indicating enhanced degradosome activity; changes in abundance for proteins involved in energy metabolism and in stress response (oxidative stress proteins and chaperones)	Van Mulders et al., 2011
*Salmonella typhimurium*	25 h space missions	MudPIT via LC-LC-MS/MS Microarray qRT-PCR	Increased virulence regulated by media composition	Differentially expressed genes involved in motility, energy production and conversion, iron utilization and uptake, ribosomal structure, and genes encoding small regulatory RNA molecules	Wilson et al., 2008
*Salmonella typhimurium*	9-day spaceflight (ISS) Simulated microgravity	Microarray Analysis Nano LC-MS/MS	Enhanced virulence in a murine infection model and extracellular matrix/biofilm accumulation	Differential expression of genes associated with ribosome structure, iron utilization/storage, periplasmic stress signalling, and biofilm formation;RNA-binding protein Hfq is identified as a global regulator involved in the response tospace environment	Wilson et al., 2007
*Salmonella enterica*	Simulated microgravity	Whole Genome Microarrays OmniGrid Array Maker, RT-PCR	Increased virulence	Differentially expressed transcriptional regulators, virulence factors, lipopolysaccharide biosynthetic enzymes, iron-utilization enzymes	Wilson et al., 2002
*Serratiamarcescens* strains LCT-SM166 and LCT-SM262	398 h space flight (Tiangong-1 space station)	Illumina HiSeq 2000 sequencer 2D-LC-MS/MS	No changes in the morphology, post-culture growth kinetics, hemolysis or antibiotic sensitivity; differences in carbon source utilization patterns	Differential expression of genes associated with glycolysis/gluconeogenesis, pyruvate metabolism, arginine and proline metabolism and the degradation of valine, leucine and isoleucine; up-regulation of genes associated with metabolism; down-regulation of *nudE* functionally associated with replication, recombination and repair; *PgaB* encoding the biofilm PGA synthesis lipoprotein; *FliE* encoding the flagellar hook-basal body complex protein; up regulation of FlgG, which encodes the flagellar basal-body rod protein	Wang et al., 2014
*Streptomyces coelicolor* A3(2)	16.5-day space mission (Tiangong-1 space station) Simulated microgravity	Microarray Agilent platform Real-time qRT-PCR analysis	Shortened life cycle; accelerated sporulation; altered secondary metabolism; increased biomass production; stronger bacteriostatic activity against *B. subtilis*	Differential expression of genes involved in morphological differentiation, aerial hyphae erection, sporulation, spore germination, cell wall structure, transport, spore structure, and development-associated secondary sigma factors; accumulation of gray spore pigment	Huang et al., 2015
*Streptococcus* mutants	Simulated microgravity	Illumina HiSeq 2500 platform	Increased killing by H_2_O_2_ compared to normal gravity control cultures	Altered expression of a number of genes located on extrachromosomal elements, as well as genes involved in carbohydrate metabolism, translation, and stress responses	Orsini et al., 2017
*Staphylococcus aureus*	Simulated microgravity	Transcriptional Affymetrix GeneChip microarray profiling	Slower growth; a novel biofilm/colonization phenotype with diminished virulence characteristics	Decreased carotenoid production, increased susceptibility to oxidative stress, and reduced survival in whole blood; alterations in metabolic pathways: carbohydrate, pyruvate, and arginine metabolism, and response to environmental stressors; down-regulation of RNA chaperone and transcriptional regulatorhfq	Castro et al., 2011
*Vibrio fischeri V. fischeri* Δhfq	Simulated microgravity	Illumina NextSeq500 platform	Changes of the growth phase transition between exponential and stationary phase	Overexpression of stress-associated genes; decrease in gene expression associated with translational activity; Δhfq mutants exhibited an increase of transcripts associated with flagellar assembly and transcriptional regulators	Duscher et al., 2018

### Carbohydrate Metabolism

The molecular mechanisms of adaptive reactions under stress conditions require additional energy and carbohydrate metabolism is crucial in generating that energy (Minic, 2015). Carbon source utilization by various microorganisms has been primarily affected during short- and long-term exposure to space conditions. In response to space environmental stress, microorganisms with great adaptability to survive successfully exhibit certain flexibility in carbon source utilization. Strains of *Serratia marcescens*, *Escherichia coli*, and *Klebsiella pneumoniae* after a long-term flight on-board of the SHENZHOU-8 spacecraft exhibited a difference in carbon source utilization (as suggested by multi-omics analysis), while their morphology or growth patterns were not affected (Li et al., 2014, 2015; Wang et al., 2014; Guo et al., 2015; Zhang et al., 2015). Strains of *S. marcescens* after spaceflight displayed a positive reaction in the sole-carbon-source utilization of D-Mannitol, D-Raffinose, and N-Acetyl neuraminic acid (Wang et al., 2014). Correspondingly, proteomic analysis showed up-regulation of the proteins of the glycolysis/gluconeogenesis pathway of *S. marcescens* grown during spaceflight, which was suggested to reflect adaptive changes of the strain aboard the spacecraft (Wang et al., 2014). The strain of *E. coli* after spaceflight showed increased carbon source utilization and 2,58-fold up-regulation of maltose regulon periplasmic protein malmM (shown in proteomic analysis), which can be explained by the increased demand for additional carbon substrates during stress adaptability to the space environment (Zhang et al., 2015). *Klebsiella pneumoniae* gained the ability to use D-Mannose after spaceflight, possibly reflecting an overall slowed down metabolism of this microorganism to better adapt to the space environment (Guo et al., 2015). Numerous *K. pneumoniae* genes differentially expressed after spaceflight were involved in carbohydrate transport and metabolism, as suggested by genomic and transcriptomic analyses (Li et al., 2014). Integrative proteotranscriptomic analysis of *Bacillus cereus* strain flown on-board of the SHENZHOU-8 spacecraft has shown that genes of glucose metabolism (*glpX* gene product, trehalose-6-phosphate hydrolase) were differentially expressed during spaceflight (transcriptomic analysis reported in Su et al., 2014). Glyceraldehyde-3-phosphate dehydrogenase (GAPDH) is one of the key enzymes in glycolytic pathway that serves to break down glucose for carbon molecules and energy. GAPDH provides an important source of NADH during glycolysis and contributes to the various regulatory functions (Sirover, 2012, 2014). Proteomic analysis showed that GAPDH was significantly up-regulated in *Bacillus pumilus* spores exposed to outer space (Vaishampayan et al., 2012). Apart from the differential expression of GAPDH under a variety of stress conditions (Nicholls et al., 2012; Hildebrandt et al., 2015), a microgravity induced up-regulation of this housekeeping protein together with pyruvate kinase and a subunit from pyruvate decarboxylase was observed in proteomic analysis of *Saccharomyces cerevisiae* after the 2-day trip on-board of the Soyuz TMA-9 vehicle (Van Mulders et al., 2011). RNAseq-based analysis of *Streptococcus* mutants displayed an alteration in early stationary-phase metabolism under the influence of simulated microgravity, and expression of phosphortransferase system genes associated with transport of carbohydrates (trehalose, mannose, glucose, mannitol, and cellobiose) was significantly increased (Orsini et al., 2017). Proteomic profiling of several fungal strains exposed to simulated Martian conditions revealed that carbohydrate metabolic functional category was among significantly over-represented biological processes (Blachowicz et al., 2019). A set of up-regulated proteins involved in carbohydrate metabolism included enzymes (e.g., isocitrate lyase AcuD, cellobiohydrolases, exo-polygalacturonase, and chitin deacetylases), which enable exposed fungal strains using an alternative carbon source and permit morphogenetic alterations in the course of growth and differentiation. The authors reported on adjustments in carbohydrate metabolism as an adaptive response to simulated Martian conditions (Blachowicz et al., 2019).

The analyzed –omics-assisted investigations indicate characteristic changes in carbohydrate metabolism of microorganisms cultivated during spaceflight and exposed to outer space. These changes are directed to restore energy balance in stress conditions and frequently serve to satisfy the increased demand in carbon source during adaptive reactions to the space environment.

### Amino Acid Metabolism

Apart from their main contribution as substrates for protein synthesis, amino acids act as signaling molecules exerting regulatory functions (Sturme et al., 2002; Rinaldo et al., 2018), and serve as indirect carbon sources through citrate cycle. Frequently observed down-regulation of enzymes involved in amino acid transport and metabolism of space-exposed microorganisms is usually associated with their arrest in growth. Among all the genes of *K. pneumonia*, *E. coli* and *B. cereus* affected during spaceflight on-board of space vehicle, functional category of amino acid transport and metabolism was the most represented (Li et al., 2014, 2015; Su et al., 2014; Guo et al., 2015; Zhang et al., 2015). The strain of *K. pneumoniae* after spaceflight characterized by increased biofilm formation was suggested to use amino acids as an indirect carbon source through TCA cycle in stress-related space conditions (Li et al., 2014). Proteomic analysis showed that most key enzymes of the TCA cycle were more abundantly represented in *Deinococcus radiodurans* cells after the exposure to UVC/vacuum conditions (Ott et al., 2017). Proteomic analysis of *S. marcescens* and *K. pneumonia* revealed that proteins involved in arginine and proline metabolism, and degradation pathways of valine, leucine, and isoleucine were down regulated after spaceflight (Wang et al., 2014; Guo et al., 2015). However, another study by Morrison et al., 2019 reported that genes of arginine biosynthesis were up-regulated during spaceflight of *B. subtilis* aboard the ISS (based on the RNA-seq analysis). Such discrepancies between different reports can be very well stated by the experimental set up of the studies. One group of the studies reported on molecular profiles during spaceflight, i.e., avoiding re-cultivation of returned samples (e.g., Morrison et al., 2019), while another group of investigations analyzed space-returned strains upon their recovery in liquid medium (e.g., Li et al., 2014). Serine hydroxymethyltransferase responsible for the enzymatic catalysis of the reversible conversion of L-serine to L-glycine was up-regulated in proteomic analysis of space-returned spores of *B. pumilus* (Vaishampayan et al., 2012). Proteomic analysis of spaceflight grown cells of *P. aeruginosa* indicated the down-regulation of ArcA, an enzyme associated with the fermentation of arginine (Crabbé et al., 2011). Proline and arginine metabolism implement in microbial mechanisms of stress survival (Zhao and Houry, 2010; Goh et al., 2011; Liang et al., 2013). Various studies have shown evidence that proline metabolism leads to increased production of endogenous reactive oxygen species (ROS) (Liang et al., 2013).

Experimental data obtained in the post-flight/post-exposure analysis suggest that amino acids metabolism is majorly down regulated in order to favor the suppressed microbial growth and proliferation. The observed suppression of proline metabolism in a number of space exposed bacterial strains can be considered as one of the microbial strategies to minimize the generation of endogenous metabolically produced ROS in order to cope efficiently with radiation-induced damage. The functional categories of cysteine and methionine metabolism, however, can be up-regulated in response to radiation component of the outer space environment (Ott et al., 2017). Methionine and cysteine are sulfur-containing amino acids, which significantly contribute to the antioxidant defense system of exposed microorganisms. Cysteine chemistry, i.e., cysteine-mediated redox signaling is important biochemical response against ROS damage (Paulsen and Carroll, 2013). Methionines located on the surface of protein structures act as effective endogenous antioxidants to defend functionally essential molecules against oxidative damage (Levine et al., 2000). In case with outer space-associated radiation exposure, activation of cysteine and methionine metabolism is one of the most obvious microbial responses to oxidative damage.

### Lipid Metabolism

Microorganisms adapt their membrane lipid composition to stressful environmental conditions by adjusting the relative amounts of different types of lipids and the degree of unsaturation of fatty acyl residues. Strains of *Bacillus horneckiae* sp. isolated from the Phoenix spacecraft were characterized by altered lipid profiles based on the results of traditional biochemical lipid analyses (Vaishampayan et al., 2010). Radiation-induced elevated generation of ROS causes multiple disintegrative disorders, including oxidative changes in lipid metabolism. The genes involved in lipid biosynthesis (enoyl-CoA hydratase/isomerase with fatty acid synthase activity) and in fatty acid metabolism were down-regulated in RNAseq-based analysis of *B. cereus* and *K. pneumoniae* strains after spaceflight (Li et al., 2014; Su et al., 2014). The authors connect it with the detected reduced growth of *B. cereus* after spaceflight as an adaptive strategy that enable the survival and maintenance of the energy status. Transcriptomic analysis of space exposed *B. subtilis* spores and *Rhodospirillum rubrum* exposed to modeled microgravity in frames of the Micro-Ecological Life Support System Alternative (MELiSSA) project showed the down-regulation of genes encoding lipid biosynthetic enzymes (Nicholson et al., 2012; Mastroleo et al., 2013). Multi-omic analyses showed that metabolic pathways associated with fatty acid metabolism, phospholipid biosynthetic process, and cellular lipid biosynthetic processes were affected in *E. coli* and *Enterococcus faecium* strains after spaceflight (Chang et al., 2013a; Li et al., 2015).

Studies of microbial biochemistry in microgravity conditions generally suggest the up-regulation of genes encoding lipoproteins and lipopolysaccharide biosynthetic enzymes (Wilson et al., 2007; Leroy et al., 2010). Several of them are associated with bacterial biofilm formation, virulence, and pathogenicity. The gene encoding rhamnosyltransferase (*rhlA*) involved in surfactant biosynthesis was found among the major virulence-associated genes of *P. aeruginosa* stimulated in spaceflight along with the accumulation of rhamnolipids under simulated microgravity conditions, which might be connected to low shear liquid sensing (transcriptomic analysis in Crabbé et al., 2011).

In summary, many of changes in lipid metabolism observed under the space environmental conditions aim to adjust the energy status toward the slowed down growth of exposed microorganisms. However, the mobilization of certain microbial lipoproteins and lipopolysaccharides is activated in spaceflight and under simulated microgravity conditions to adapt cell-cell contacts and communication toward the low-shear growth environment.

## Membrane-Associated Processes

The destructive effect of space vacuum (10^−7^ to 10^−4^ Pa) triggers cellular integrity of space-exposed microorganisms, influencing numerous processes in membrane apparatus of microbial cell (Horneck et al., 2010). Among them are rearrangements of lipid bilayers, changes in membrane fluidity and permeability, and alteration of membrane bound enzymatic activities. Cell membrane that carries a function of physical barrier and protect the cell from extracellular environment can be affected in conditions of microgravity, causing the altered uptake or excretion rates.

### Cell Envelope Biosynthesis and Maintenance

Microbiological and biochemical analysis of the survival and behavior of *C. metallidurans* in the MESSAGE-1 flight experiment by means of flow cytometry-assisted analysis of cell physiology and proteomic profiling revealed a minor damage of cell membrane; the remaining viable cells acquired a higher membrane potential than the ground control cells (Leys et al., 2009). The genes involved in the cell wall/membrane/envelope biogenesis of *E. faecium* were among the differentially expressed genes with the greatest change in expression after flight on the SHENZHOU-8 spacecraft, as suggested by comparative transcriptomic and proteomic analyses. Comparative genomic analysis of returned after spaceflight *E. faecium* strain revealed mutation of the *arpU* gene associated with cell wall growth, which in turn may affect the expression of molecular players responsible for cell wall and membrane biogenesis of *E. faecium* (Chang et al., 2013a). The post-flight transcriptomic and proteomic studies of *E. coli* revealed the up-regulation of the envelope stress induced periplasmic protein Spy and the *yfbE* gene encoding predicted pyridoxal phosphate-dependent enzyme with regulatory functions in cell wall biogenesis (Li et al., 2015; Zhang et al., 2015). A number of differentially expressed genes involved in cell wall and spore structure were described microarray-based analysis of *Streptomyces coelicolor* during spaceflight and simulated microgravity (Huang et al., 2015). Modeled microgravity affected gene groups of *Salmonella* involved in type III secretion, lipopolysaccharides, and cell wall synthesis (microarray-based analysis in Wilson et al., 2002). Proteomic analysis showed that *D. radiodurans* grown in simulated microgravity showed an increased abundance of cell envelope-associated proteins (Ott et al., 2019a). Cell envelope biosynthesis and maintenance was triggered in multi-omics analysis of *R. rubrum* by spaceflight and simulated space environment (the top 20 of the most induced genes) (Mastroleo et al., 2009, 2013). Cell envelope of *B. subtilis* exposed to 1.5 years of simulated Martian and space conditions was massively affected as suggested by a comprehensive transcriptomic analysis with a number of up-regulated membrane and cell envelope stress proteins (SigV regulon) (Nicholson et al., 2012).

A number of spaceflight induced alterations associated with cell envelope, e.g., a thickened cell envelope and intensive vesiculation (Zea et al., 2017), are quick stress responses to enhance microbial adaptation rates and regulate the level of protein accumulation in the cell envelope. These autonomous stress responses are mediated by means of altered cell envelope protein machinery, allowing exposed cells to export stress products (e.g., damage or misfolded proteins) and to achieve alleviated stress response.

### Transport

The effects of simulated and real microgravity on microbial behavior and metabolism in liquid cultures aboard spacecraft are most likely mediated by alterations of extracellular environment. The response of the cell to changes in the extracellular environment includes a cascade of cellular transport events that operate nutrient uptake, cellular waste disposal, solute transport and quorum-sensing signaling. The Suf along with other ABC membrane transporters were identified as differentially abundant in *S. typhimurium* in proteomic response to cultivation aboard spacecraft (Wilson et al., 2008). Spaceflight significantly affected the transport machinery of *E. coli*, up-regulating a number of transporters, as shown in multi-omics analysis (Li et al., 2015). The multi-omics based comparison of the strains after spaceflight and the control strains of *E. coli*, *K. pneumonia*, and *R. rubrum* showed that ontological categories “transmembrane transporter activity” were overrepresented among all differently expressed genes and differently abundant proteins during spaceflight (Mastroleo et al., 2009; Li et al., 2014, 2015). The genes encoding for multidrug efflux and arsenite membrane-bound transporters were 3 to 4 fold overexpressed in transcriptomic analysis of spores of *B. subtilus* exposed to simulated Mars conditions or/and real space (Nicholson et al., 2012). The genes encoding for probable metal-transporting P-type ATPase and dctA C4-dicarboxylate transport protein were overexpressed as suggested by post-flight transcriptomic analysis of *P. aeruginosa* (Crabbé et al., 2011). Microarray analysis identified that the group of membrane transport genes belongs to low-shear modeled microgravity (LSMMG) regulon of *Salmonella* and the gene *sbmA* encoding ABC superfamily transporter was up-regulated during spaceflight (Wilson et al., 2007). Post-flight proteotranscriptomic analysis of *R. rubrum* revealed up-regulated genes that are involved in solute transport and osmotic regulation, and are probably related to oxidative stress (Mastroleo et al., 2009). Antibiotic-producing *S. coelicolor* in conditions of simulated and real space microgravity displayed a significant up-regulation of several transporters which contribute to its enhanced bioactivity and BldK ABC transporter complex which is essential for aerial mycelium formation (microarray-based analysis in Huang et al., 2015).

Due to the lack of gravity-driven convective flows, extracellular mass transport becomes essentially limited to diffusion under the conditions of spaceflight and simulated microgravity. Cells cultured in liquid medium during spaceflight may experience a deficiency in oxygen and nutrients availability (Zea et al., 2016), which reflects altered transport functions. The elevated transport machinery of microgravity-affected microorganisms aims to facilitate not only nutrient uptake, but also cellular waste removal, distribution of solute and trafficking of quorum-sensing signaling molecules.

### Chemotaxis, Cell Motility

Microgravity in conditions of spaceflight affects extracellular fluid properties, this way altering relationship between microbial cell and extracellular environment (Horneck et al., 2010). The involvement of flagellar apparatus and chemotaxis machinery after spaceflight and real space exposure has been detected applying –omics assisted analyses of several bacterial strains. 5.1-fold up-regulation of the *fliL* gene required for flagellar formation was indicated in the transcriptomic response of space-exposed spores of *B. subtilis* (Nicholson et al., 2012). The *flgG* gene encoding the flagellar basal-body rod protein was up-regulated in transcriptomic response of *S. marcescens* to spaceflight conditions, while down-regulation was shown in the same study for the *FliE* gene that encodes the flagellar hook-basal body complex protein (Wang et al., 2014). In addition, many *K. pneumoniae* genes affecting cell motility were differentially expressed in the strain exposed to simulated space condition (Li et al., 2014). The expression of flagellar assembly genes in LSMMG conditions was also increased in the transcriptome of the mutualistic bacterium *Vibrio fischeri* (Duscher et al., 2018). Flagellar assembly and bacterial chemotaxis were the most significantly enriched functional categories among all affected genes differently expressed by *E. coli* during spaceflight, e.g., genes encoding bacterial flagellin and methyl-accepting chemotaxis protein II (Li et al., 2015). Transcriptomic response of *R. rubrum* to spaceflight and simulated microgravity indicated the induced expression (upregulated > 2.6-fold) of the gene encoding for Flagellar hook-associated protein 2 (FliD filament cap protein) (Mastroleo et al., 2009). Genes of *S. typhimurium* involved in motility and chemotaxis response were identified as differentially expressed in response to spaceflight cultivation (Wilson et al., 2007). Transcriptomic and proteomic analyses of *P. aeruginosa* exposed to spaceflight conditions revealed that several chemotaxis transducers were up-regulated in response to spaceflight (Crabbé et al., 2011).

The reported effects of simulated microgravity and spaceflight on the various physiological properties of microorganisms are associated with the potential disruption of the quiescent extracellular environment. The molecular components of bacterial motility and chemotaxis response are activated in microgravity-exposed microorganisms in order to gain a balanced connection between the cell and its environment. The induced flagellar action results in mixing of the local fluid surrounding of microbial cell and removal of the cell from its quiescent location. The induction of molecular machinery responsible for motility and chemotaxis can locally influence the correct redistribution and availability of nutrients, substrates, solutes and other biologically active molecules at the extracellular environment level.

### Energy Production and Conversion

In line with the increased demand in energy needed to cope with stress in space environment, there are several post-flight –omics assisted observations of altered membrane bioenergetics and electron transport chains with elevated abundances of electron transfer proteins and ATP synthase. Comparative proteomic analysis of *B. pumilus* spores long-termly exposed to a variety of real space conditions at the ISS in the EXPOSE facility revealed the up-regulation of the alpha-ketoglutarate decarboxylase enzyme which is associated with menaquinone biosynthesis and involved in the membrane-associated electron transport system (Palaniappan et al., 1994; Vaishampayan et al., 2012). Further proteomic studies with space-exposed *B. pumilus* showed that the altered protein abundances in the category of energy metabolism might be a microbial strategy to better cope with stressful environments (Chiang et al., 2019). Post-flight proteotranscriptomic analysis of *R. rubrum* identified significant up-regulation of multiple clusters of genes relevant to energy production and conversion (succinate dehydrogenase, ubiquinone oxidoreductase) (Mastroleo et al., 2009). The *hydN* gene encoding Fe-S center-bearing protein responsible for electron transport from formate to hydrogen together with a set of *S. typhimurium* genes involved in the formation of the Hyc hydrogenase (respiratory enzyme of H_2_-uptake) were differentially expressed in response to spaceflight cultivation (Wilson et al., 2007). The gene *hppA* encoding membrane-bound proton-translocating pyrophosphatase and five F_0_F_1_ ATP synthase subunits were up-regulated after spaceflight of *R. rubrum* (Mastroleo et al., 2009). The subunits of ATP synthase of microgravity-exposed *S. cerevisiae* have been differentially regulated during short-term spaceflight (Van Mulders et al., 2011). The down-regulation of CcoP2, a cytochrome with high affinity for oxygen, has been observed in proteomic analysis of the *P. aeruginosa* cells grown in spaceflight (Crabbé et al., 2011). This cytochrome is not active in the anaerobic lifestyle of *P. aeruginosa*, but is induced under microaerophilic conditions (Crabbé et al., 2011). The authors reported that *P. aeruginosa* adopted an anaerobic mode of growth during spaceflight, and switched to anaerobic metabolism, which was accompanied by the down-regulation of CcoP2 under oxygen-limiting conditions. Spaceflight induced mainly genes involved in anaerobic metabolism of this pathogen and anaerobic respiration occurred through denitrification, i.e., in the presence of the alternative electron acceptor nitrate or nitrite (Crabbé et al., 2011).

Microbial adaptation to spaceflight conditions may lead to alterations associated with energy sources utilization. Hereby, a corresponding molecular pull of energy-converting enzymes responsible for a switch from one energy source to another is affected in conditions of spaceflight and simulated microgravity. Energy management also facilitates the survival of outer space exposed microorganisms by modulating electron transfer proteins.

### Iron Utilization and Uptake

Iron as the most abundant transition metal in biological systems is incorporated into protein cofactors and plays important regulatory, redox and catalytic roles in microbial world. Prokaryotes have evolved powerful iron assimilation and storage systems, which supply sufficient iron for growth and metabolism. Microorganisms show an enormous diversity and abundance of iron-dependent redox proteins, which majorly harbor iron within hemes and in the form of iron–sulfur (Fe–S) clusters. Bacteria and Archaea enormously depend on these two classes of cofactors for their energy metabolism. Iron availability influences the expression of bacterial genes encoding high-affinity iron uptake pathways and, in pathogenic microorganisms, virulence determinants (Butt and Thomas, 2017). The metal-specific repressor Fur (ferric uptake regulator) (Lee and Helmann, 2007) appears to be a very global and well-conserved regulator, which participates in regulation of iron uptake and homeostasis in bacteria. Fur coordinates the expression of various genes and acts as an iron-responsive, DNA-binding repressor protein (Hantke, 1987; Lee and Helmann, 2007). The Fur protein employs Fe(II) as a cofactor and binds to a “Fur box” with the palindromic consensus sequence GATAATGATAATCATTATC in the promoters of iron-regulated genes, resulting in repression of these genes, while under low-iron conditions, the Fur protein is released from this operator site and transcription takes place. The Fur repressor is involved in the transcriptional control of the operons encoding the pathways for the production of the siderophores (responsible for delivering the iron into the cells), virulence-associated genes, the manganese- and iron-containing superoxide dismutase genes and in the *fur* gene autoregulation (Hall and Foster, 1996; Lee and Helmann, 2007).

The Fur protein and iron utilization/storage system were shown to play a role in the *Salmonella* ground-based LSMMG (Wilson et al., 2002) and the spaceflight-induced multi-omics molecular responses (Wilson et al., 2002, 2008). LSMMG induces acid resistance in *Salmonella* and Fur was shown to be essential in this process. The *fur* mutant strain of *Salmonella* did not demonstrate any detectable acid resistance to be induced by LSMMG (Wilson et al., 2002). Furthermore, several genes involved in iron metabolism were induced (*fepD* encoding for ferric enterobactin transporter, *STM1537* encoding for Ni/Fe-hydrogenase1 b-type cytochrome subunit, *hscB* responsible for assembly of Fe-S clusters) and down regulated (*feoB* encoding for ferrous iron transport protein, *yliG* putative Fe-S oxidoreductase, *sufC*, *sufS*) by LSMMG, as indicated by whole genome microarray analysis (Wilson et al., 2002). Additionally, the authors mention a number of potential Fur-binding sites that were located upstream of several different LSMMG regulated genes. The Fur-binding site-associated genes regulated by LSMMG included those identified to be regulated by Fur (*fepD*, *sufC*, *sufS*, and *feoB*) and those not earlier described as Fur regulated (Wilson et al., 2002). Wilson et al. (2007, 2008) reported that several molecular components of iron utilization/storage system were altered during spaceflight. Proteomic analysis showed that the abundances of Fur, iron-dependent alcohol dehydrogenase, bactoferrin, and electron transport protein with Fe-S center were increased after spaceflight, while a number of other proteins involved in iron utilization and uptake (e.g., cytoplasmic ferritin, siderophore receptor TonB, Fe-S cluster formation protein, and ferric enterobactin receptor) were underrepresented during growth of *S. typhimurium* in spaceflight (Wilson et al., 2007, 2008). These findings indicate the involvement of Fur in transmitting the LSMMG and spaceflight induced signals.

Proteotranscriptomic analysis of *P. aeruginosa* showed that the gene *bfrB* encoding bacterioferritin was down-regulated in cells grown on-board (Crabbé et al., 2011). Apart from the primarily function of iron storage, bacterioferritins participate in defense against oxidative stress and radical damage (Velayudhan et al., 2007). The down-regulation of *bfrB* in this case may be linked to the anerobiosis switch of *P. aeruginosa* during spaceflight (Crabbé et al., 2011). 4 to 6-fold down-regulation of several genes encoding putative iron (III) dicitrate transporting proteins was shown in transcriptomic analysis of spores of *B. subtilus* exposed to 1.5 year to real space conditions (Nicholson et al., 2012). The analysis of differential gene expression of *R. rubrum* in MESSAGE 2 spaceflight experiment (Mastroleo et al., 2009) revealed the up-regulation of the Fur repressor, which connects cellular iron status to oxidative stress by scavenging iron (Lee and Helmann, 2007), and the down-regulation of hfq, which negatively controls *fur* expression in *E. coli* (Vecerek et al., 2003). During MESSAGE 2 experiment the genes involved in the iron acquisition and redox balance, e.g., ferredoxin (Rru_A0077), Fe-S cluster-related gene (Rru_A1069), *dps*-related gene (Rru_A1499), superoxide dismutase (Rru_A1760) and bacterioferritin *bfr* (Rru_A2195) were induced (Mastroleo et al., 2009).

The analyzed –omics based investigations indicate that modeled microgravity and spaceflight conditions operate molecular iron-binding elements for their regulatory and oxidative stress pathways, encompassing a novel environmental signal.

## Genetic Machinery

**FIGURE 2:**
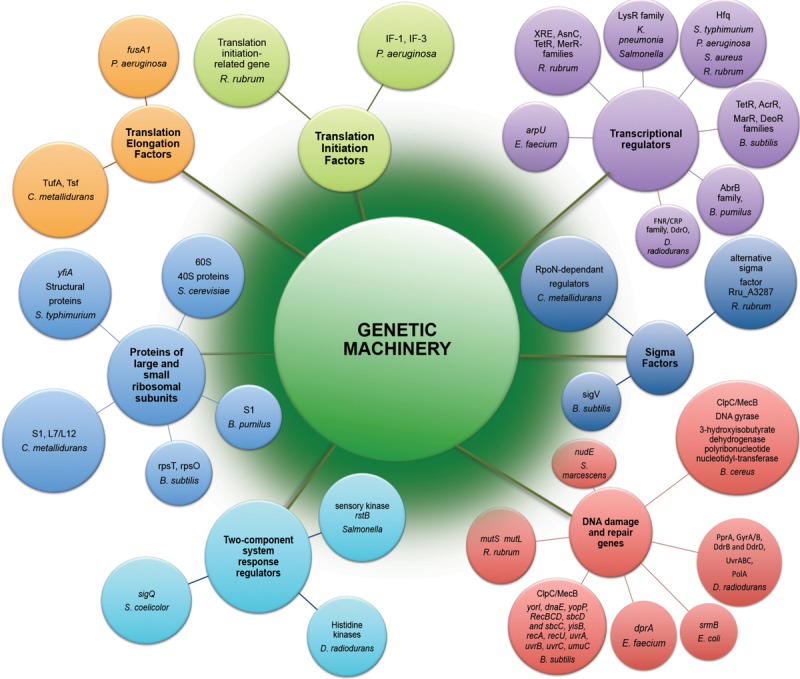
Genetic machinery implicated in molecular response to exposure of microorganisms to the outer space environment. Represented are differently expressed genes and differently abundant proteins of microorganisms exposed to real and simulated space conditions (Table 1) revealed by means of –omics assisted methodological approaches.

The potential effects of space radiation target genetic stability of space-traveling microorganisms. Both by direct damage to DNA and indirect consequences due to ROS generation, radiation component of space environment affects genetic machinery of exposed microorganisms (Figure 2), causing a wide variety of changes starting from gene expression patterns (Table 1) and frequently leading to space-induced DNA mutagenesis (Horneck and Rabbow, 2007; Moeller et al., 2012). Global alterations in gene expression at the translational and transcriptional levels induced by adaptation of exposed microorganisms to radiation- and microgravity-filled space environment have been confirmed by proteomic and transcriptomic analyses (Figure 2), while genomics techniques revealed a number of mutant microbial strains after spaceflight (Vaishampayan et al., 2010; Chang et al., 2013a, b; Li et al., 2014; Zhang et al., 2015).

### Translation, Ribosomal Structure, and Biogenesis

The multi-omics post-flight observations of *K. pneumonia*, *E. faecium* and *R. rubrum* cultivated on-board spacecraft revealed that the functional category “translation, ribosomal and biogenesis” was one of most highly represented category among all the up-regulated genes (Mastroleo et al., 2009; Chang et al., 2013a; Guo et al., 2015). More than 20 ribosomal protein-encoding genes of *R. rubrum*, 1 translation initiation-related gene, and 2 translation elongation-related genes have been found up-regulated in transcriptomic response to spaceflight (Mastroleo et al., 2009). Ribosomal apparatus of *R. rubrum, P. aeruginosa*, *S. typhimurium*, *B. pumilus*, *B. subtilis*, *B. cereus*, *S. cerevisiae* and *C. metallidurans* has been also massively affected in conditions of modeled microgravity (Wilson et al., 2002; Leroy et al., 2010; Mastroleo et al., 2013), spaceflight (Leys et al., 2009; Su et al., 2014; Zhang et al., 2015) and in real space conditions (Nicholson et al., 2012; Vaishampayan et al., 2012) with a number of down-regulated ribosomal genes and proteins as suggested by the multi-omics based analysis. Of 115 genes of *P. aeruginosa* that were down-regulated during spaceflight, 40 were involved in the synthesis of ribosomes (Crabbé et al., 2011). The *P. aeruginosa* genes encoding translational elongation factor (*fusA1*) and translation initiation factors IF-1 and IF-3 were down-regulated in transcriptomic response to spaceflight cultivation (Crabbé et al., 2011). Several enzymes involved in ribosomal protein translation (translation elongation factors TufA, Tsf) were more abundantly represented in proteomic analysis of cells of *C. metallidurans* after the exposure to spaceflight within the MESSAGE experiments (Leys et al., 2009).

The elevated level of translation-related genes and proteins (Figure 2) can support the higher abundances of proteins related to the metabolic and stress responses in microorganisms exposed to space conditions.

### Transcription

One of the most noteworthy and challenging matters in the understanding of the effects of space environmental factors is the identification of global master regulators of space-induced response (Figure 2), that serve to globally reprogram microbial physiology in order to permit the adaptation of microorganisms to the space environment.

Transcriptomic analysis of *R. rubrum* (Mastroleo et al., 2009) cultivated aboard spacecraft, *B. subtilis* (Nicholson et al., 2012) and *B. pumilus* (Vaishampayan et al., 2012) spores exposed to outer space conditions and *Salmonella* in LSMMG conditions (Wilson et al., 2002) revealed a wide repertoire of transcriptional regulators involved in response to a long-term exposure to outer space. Interestingly, genomic analysis of *E. faecium* (Chang et al., 2013a) and *K. pneumonia* (Guo et al., 2015) mutant strains after spaceflight identified point mutations in genes encoding transcriptional regulators, including *arpU* gene that plays a role in cell wall growth and division by controlling the muramidase-2 export (Chang et al., 2013a). Remarkably high in *B. subtilis* spores exposed to simulated and real space conditions was 24- to 29-fold up-regulation of *fruR* transcriptional repressor of *fru* operon (DeoR family), which is implicated in metabolism of carbohydrates triggered in majority of space exposed microorganisms (Nicholson et al., 2012). Transcriptional regulator of AbrB family responsible for the repression of various starvation-induced differentiation processes was up-regulated in proteomic analysis of space-exposed spores of *B. pumilus* (Vaishampayan et al., 2012). The elevated transcript levels of several of *B. subtilis* master regulators were revealed in transcriptomic analysis; the stimulation of these master stress-responsive genes was higher in space-exposed spores of *B. subtilis*, than in spores exposed to simulated Mars environment (Nicholson et al., 2012).

Apart from the transcription factors, the *rstB* gene encoding a putative membrane sensory kinase was identified in microarray analysis of *Salmonella* LSMMG response (Wilson et al., 2002), acting as part of a two-component system to accomplish signal transduction and reprogram the cell physiology in conditions of LSMMG. Multi-omics analyses of *B. subtilis*, *C. metallidurans*, *R. rubrum*, and *S. coelicolor* identified several sigma factors involved in response to the space conditions (Leys et al., 2009; Mastroleo et al., 2009; Nicholson et al., 2012; Huang et al., 2015). Proteometabolomic analysis of *D. radiodurans* exposed to simulated space conditions showed the involvement of the transcriptional regulator of FNR/CRP family and DdrO, the transcriptional regulator of HTH_3 family in response to UVC/vacuum combined stress (Ott et al., 2017). The RNA chaperone and global regulator Hfq (Sauter et al., 2003; Vogel and Luisi, 2011; Sauer, 2013) has been directly involved in mechanisms of spaceflight and LSMMG microbial responses (Wilson et al., 2007, 2008; Mastroleo et al., 2009; Castro et al., 2011; Crabbé et al., 2011) (Figure 2). Apart from the highly abundant transcriptional regulator of FNR/CRP family, specific histidine kinases might be also involved in the regulation of vacuum stress response in *D. radiodurans* as suggested by proteomic analysis (Ott et al., 2019b).

The adaptation of microorganisms to the spaceflight conditions and microbial survivability in the outer space environment is realized in the complex of responses, which are controlled under the regulation of specific genetic elements – master regulators of transcriptional response. The –omics based analyses revealed a number of upstream transcriptional regulators which affect downstream gene expression activity in response to space environmental parameters.

### DNA Replication, Recombination, and Repair

Extremophilic microorganisms in the outer space environment tolerate radiation stress and cope with radiation-induced DNA damage by means of their DNA repair molecular machinery (Figure 2). A comparative multi-omic analysis of *B. cereus* and *E. faecium* strains after spaceflight (Chang et al., 2013a; Su et al., 2014) and transcriptomic analysis of *B. subtilis* long-termly exposed to simulated Martian and real outer space conditions (Nicholson et al., 2012) showed that the functional category of DNA replication, recombination, and repair was among the most abundantly represented categories of proteins and genes differently transcribed in comparison with a control ground strains. The DNA mismatch repair genes *mutS* and *mutL* were up-regulated in transcriptomic response to space flight of *R. rubrum* (Mastroleo et al., 2009). Putative replicative DNA helicase (*yorI*) and DNA polymerase III alpha-subunit 3 (*dnaE*) were 3- to 14-fold up-regulated in space-exposed and simulated Mars-exposed *B. subtilis* spores (Nicholson et al., 2012). The *srmB* gene encoding DNA helicase was up-regulated in *E. coli* under simulated microgravity conditions (Arunasri et al., 2013). A number of other up-regulated genes associated with DNA repair and recombination were identified in transcriptional response of *B. subtilis* to real and simulated space conditions (Figure 2) (Nicholson et al., 2012). Especially remarkable was 44-fold up-regulation of the gene *umuC* (*yqjW*) encoding Y-family DNA polymerase, responsible for translesion bypass DNA repair in space-exposed *B. subtilis* spores (Nicholson et al., 2012).

A recent proteomic analysis of *D. radiodurans* exposed to simulated space conditions indicated the up-regulation of DNA damage response proteins PprA, GyrA/B, DdrB and DdrD in UVC/vacuum-affected cells, along with high constitutive RecA levels (Ott et al., 2017). Moreover, UVC/vacuum stress conditions stimulated a number of proteins involved in detoxification process and aimed to remove damaged nucleotides from *D. radiodurans* cells (e.g., UvrB, a helixase subunit of the DNA excision repair endonuclease complex, MutT/nudix, and MutS2 families proteins involved in mismatch excision repair). The abundances of the proteins (recQ and ruvABC) responsible for recombinational DNA repair and the Mrr restriction system protein were also significantly increased (Ott et al., 2017). Furthermore, an increase in proteins of the UvrABC nucleotide excision repair machinery and polymerase PolA was observed during the 1st hours of recovery *D. radiodurans* after 90 days of exposure to simulated vacuum conditions of Low Earth Orbit (proteomic-based analyses in Ott et al., 2019b).

Obviously, “space travelers” utilize a striking up-regulation of DNA damage response genes and proteins as an important strategy to cope with space radiation induced DNA damage. Various DNA damage response systems react in order to handle with the stressful situation caused by spaceflight, outer space exposure and simulated space conditions.

## General Cellular Responses

Among other characteristics of the influence of the space environment on microbial cell behavior and physiology are numerous stress responses, altered tellurium resistance, biofilm formation, sporulation and virulence of several opportunistic and obligate pathogenic microorganisms.

### General Stress Response

**FIGURE 3:**
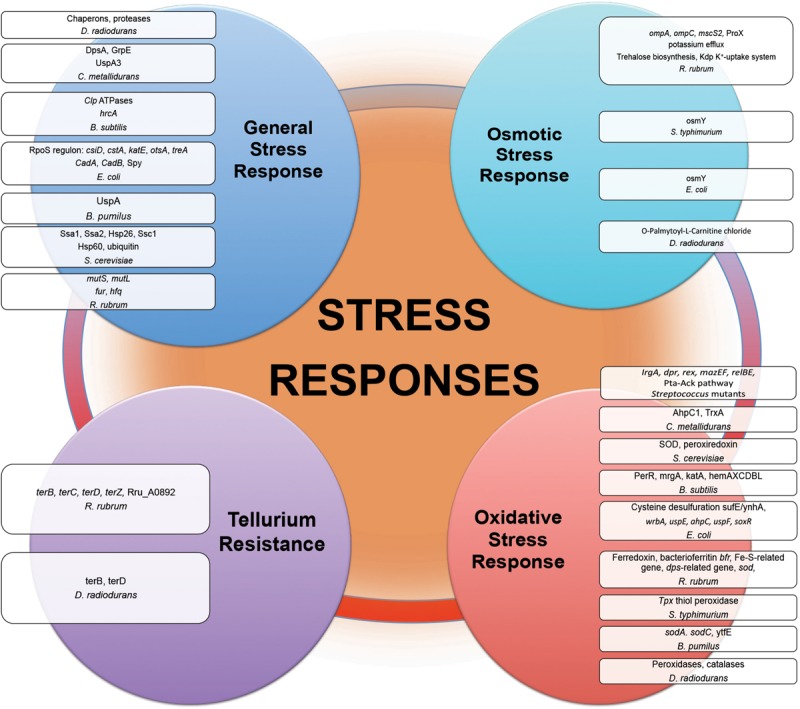
Stress responses experienced by microorganisms in outer space real and simulated conditions. Proteins and genes of numerous stress responses with altered abundance and expression after exposure of microorganisms (Table 1) to the outer space real and simulated environment.

Proteins of general stress response function to protect cells, restore damage to cellular and molecular structures (e.g., DNA, the cell envelope, and proteins), and to provide microorganisms the ability to recover from the stress they experience. Overexpression of stress response genes was observed by real-time-PCR in *E. coli* under modeled reduced gravity conditions (Vukanti and Leff, 2012), in proteomic response of *C. metallidurans* grown aboard spacecraft (Leys et al., 2009) and in multi-omics analysis of *R. rubrum* exposed to spaceflight and simulated microgravity (Mastroleo et al., 2009, 2013) (Figure 3). Induction of stress-responsive proteins with the function of stress/protein folding and oxidative stress has been shown in proteomic analysis of *S. cerevisiae* aboard Soyuz TMA-9 (Van Mulders et al., 2011). Systems for monitoring protein quality within the cell devoted to control protein damage and misfolding implement in transcriptomic-assisted stress response of *B. subtilis* to real space and simulated Martian conditions (Nicholson et al., 2012) and proteomic response of *S. cerevisiae* to microgravity conditions during short-term spaceflight (Van Mulders et al., 2011) (Figure 3). The increased abundances of a number of chaperons and proteases were observed in proteomic response of *D. radiodurans* to UVC/vacuum (Ott et al., 2017), implying the involvement of proteolytic regulation and quality monitoring in response to simulated space conditions. An increased abundance of *S. cerevisiae* ubiquitin in microgravity conditions was detected in proteomic analysis, indicating an increased degradosome activity and suggesting that cells of exposed microorganisms are prone to experience protein damage and/or misfolding as a consequence of exposure to space parameters (Van Mulders et al., 2011).

Various heat shock proteins and numerous chaperone proteins are frequently more abundantly represented in exposed microorganisms. Being involved in diverse metabolic processes and responsible for protein folding, these proteins can prevent or reverse protein misfolding. By binding to proteins, which are misfolded and damaged in response to the outer space environmental stresses, these molecular chaperones can direct the misfolded proteins to the associated proteases for degradation. The elevated level of different types of proteases under the influence of space-associated environmental factors indicates the involvement of quality monitoring and proteolytic regulation in response to outer space environmental stress.

### Oxidative Stress Response

Comparative -omics assisted investigations revealed various universal ROS scavengers, e.g., superoxide dismutase (SOD), and redox active proteins (peroxiredoxin, thiol peroxidase, thioredoxin, catalase, sulfoxide reductase MsrA) induced in “real” space-exposed spores of *B. pumilus* and *B. subtilis*; *E. coli*, *R. rubrum*, and *S. typhimurium* on-board of spacecraft, and *D. radiodurans* exposed to space simulating conditions, manifesting the up-regulation of antioxidant defense mechanisms during long-term spaceflight (Figure 3) (Wilson et al., 2008; Mastroleo et al., 2009; Nicholson et al., 2012; Vaishampayan et al., 2012; Ott et al., 2017). The PerR regulon consisting of a cluster of genes, which are stimulated in response to oxidative stress, was activated in transcriptomic analysis of *B. subtilis* spores long-termly exposed to ionizing radiation, vacuum, and extreme desiccation in outer space (Nicholson et al., 2012). Being essential for the resistance to oxidative stress, SOD (Fukai and Ushio-Fukai, 2011) by scavenging the superoxide (O^2–^) radical, provides an efficient antioxidant defense in conditions of outer space. In opposite to its bacterial counterpart under the long-term influence of real space factors, SOD from spaceflight microgravity-exposed yeast *S. cerevisiae* along with the other oxidative stress protein peroxiredoxin was down-regulated in proteomic analysis after short-time space flight (Van Mulders et al., 2011). Together with the suppression of enzymes involved in oxidative metabolism, SOD down-regulation in microgravity conditions may indicate the shift of *S. cerevisiae* to anaerobiosis during spaceflight. *P. aeruginosa* has also shown an adaptation to anaerobic mode of growth during spaceflight with a number of up-regulated genes involved in anaerobic metabolism (Crabbé et al., 2011). The increased abundances of ROS scavenging proteins, e.g., peroxidases and catalases, were observed in proteomic analysis of *D. radiodurans* exposed to simulated Low Earth Orbit vacuum conditions (Ott et al., 2019b). The stress response genes contribute to the increased antibiotic tolerance of *E. coli* in microgravity during spaceflight aboard the ISS, as suggested by RNA-Seq assisted analysis (Aunins et al., 2018).

Mining data from –omics-assisted studies clearly shows that antioxidant defense mechanisms are important part of microbial responses to cope with space-induced oxidative damage.

### Osmotic Stress Response

Results of post-flight proteotranscriptomic analysis of *E. coli* and *R. rubrum* indicated the altered expression of a number of genes involved in solute transport and osmotic regulation (Figure 3) (Mastroleo et al., 2009; Li et al., 2015; Zhang et al., 2015). The osmoprotectant glycine betaine transporter ProX of *R. rubrum* has been indicated as one of the very few proteins up-regulated in proteomic response under the conditions of simulation of ISS-ionizing radiation (Mastroleo et al., 2009). Osmotically inducible proteins were among up-regulated proteins in proteomic analyses of *S. typhimurium* and *E. coli* after spaceflight (Wilson et al., 2007; Zhang et al., 2015). *S. typhimurium* grown in conditions of modeled microgravity displayed increased resistance to multiple environmental stresses, including resistance to osmotic stress (Wilson et al., 2002). Notably, metabolomics analysis of cells of *D. radiodurans* exposed to simulated space conditions revealed the elevated level of a palmitoyl-derivative of carnitine, a quaternary amine compound with various physiological effects (Ott et al., 2017). Carnitine is a compatible solute and important osmoprotectant, which can augment thermotolerance, cryotolerance and barotolerance, thus influencing bacterial survival in extreme conditions (Meadows and Wargo, 2015). This compatible solute can help to cope with osmotic stress as a damaging desiccation effect of vacuum (Horneck et al., 2010; Frosler et al., 2017) by binding additional water molecules, stabilizing proteins and cell membranes, and thus blocking complete desiccation of the cell. The up-regulation of O-Palmitoyl-L-Carnitine chloride was suggested to play a role in the defense of *D. radiodurans* against combined stress conditions of UVC and vacuum (Ott et al., 2017).

Space environmental parameters inflict a cellular stress state that has the characteristics similar to an osmotic stress. Along with an activation of cell wall-associated machinery and integrity pathways, the production of osmoprotective compounds (e.g., compatible solutes) that increase the osmotolerance serve as a microbial strategy to cope with outer space environmental stressors.

### Tellurium Resistance

Tellurium resistance does not necessarily constitute a distinct resistance determinant in microorganisms, but it may represent a resulting effect of a specific metabolic function, such as oxidative stress response (Taylor, 1999; Chasteen et al., 2009). Post-flight analysis of tellurium resistance of *R. rubrum* sent to the ISS in frames of MELiSSA project and in conditions of modeled microgravity indicated an enhanced expression of genes involved in tellurium resistance in transcriptomic analysis (Figure 3) (Mastroleo et al., 2009). The tellurium resistance proteins TerB and TerZ are associated with the resistance of *E. coli* against various damaging agents (e.g., heavy metal ions and UV radiation), and contribute to the preservation of the intracellular reducing environment, probably by directly reversing disulfide bonds (Slade and Radman, 2011). Oxidative stress-responsive proteins within tellurium resistance operon in *D. radiodurans* were found to be up-regulated immediately after gamma-irradiation (Slade and Radman, 2011). Moreover, TerB and TerD were up-regulated in proteomic response of *D. radiodurans* exposed to UVC/vacuum simulated space conditions (Ott et al., 2017). The enhanced tellurium resistance of exposed microorganisms can be either linked to another metabolic function or a part of metal sensing stress response system.

### Sporulation

Frequently, space microbiology concentrates on spore-forming bacteria such as *Bacillus* due to the remarkable resistance of their spores to harsh conditions (Nicholson et al., 2000). Antibiotic-producing and spore-forming *Streptomyces* also represent additional interest due to the modulation of secondary metabolites production in space environment. The sporulation process of microgravity exposed *S. coelicolor* was intensified during spaceflight, and increased accumulation of the gray spore pigment and the fastened transition from aerial hyphae to mature spores were observed on-board the SHENZHOU-8 spacecraft (Huang et al., 2015). Global transcriptional analysis revealed the differential expression of genes involved in morphological differentiation and development of streptomycetes, which are mainly linked to aerial hyphae erection, sporulation, spore germination, cell wall structure, spore structure, and development-associated secondary sigma factors (Huang et al., 2015).

Proteotranscriptomic analysis of *B. cereus* after short-term spaceflight showed the down-regulation protein MreB, which determines rod-shape of *B. cereus* (Su et al., 2014). A number of genes involved in sporulation were induced in transcriptional analysis during germination of *B. subtilis* spores after the exposure to simulated Martian and real space conditions (Nicholson et al., 2012). Significant up-regulation of the *cgeAB* operon involved in maturation of the outermost spore layer together with increased transcript levels of genes encoding minor and major acid-soluble spore proteins has been observed in this study. The genes *cotG*, *cotT*, and *cotVWXY* encoding protein components of the spore coat, the *safAcoxA* operon responsible for spore morphogenesis and spore cortex formation, and *rapAphrA* operon which controls initiation of sporulation were also significantly up-regulated in *B. subtilis* (Nicholson et al., 2012). Comparative proteomics analysis indicated that the outer spore coat protein A was modulated in spores of *B. pumilus* exposed to the UV- space–and UV-Mars–conditions (Vaishampayan et al., 2012).

Sporulation is a widely used strategy that helps to various spore-forming microorganisms to adapt and survive in harsh conditions of outer space. –Omics-assisted investigations helped to reveal the various molecular components involved in sporulation across different microbial species after exposure to the space environment.

### Pathogenicity, Virulence, and Biofilm Formation

**FIGURE 4:**
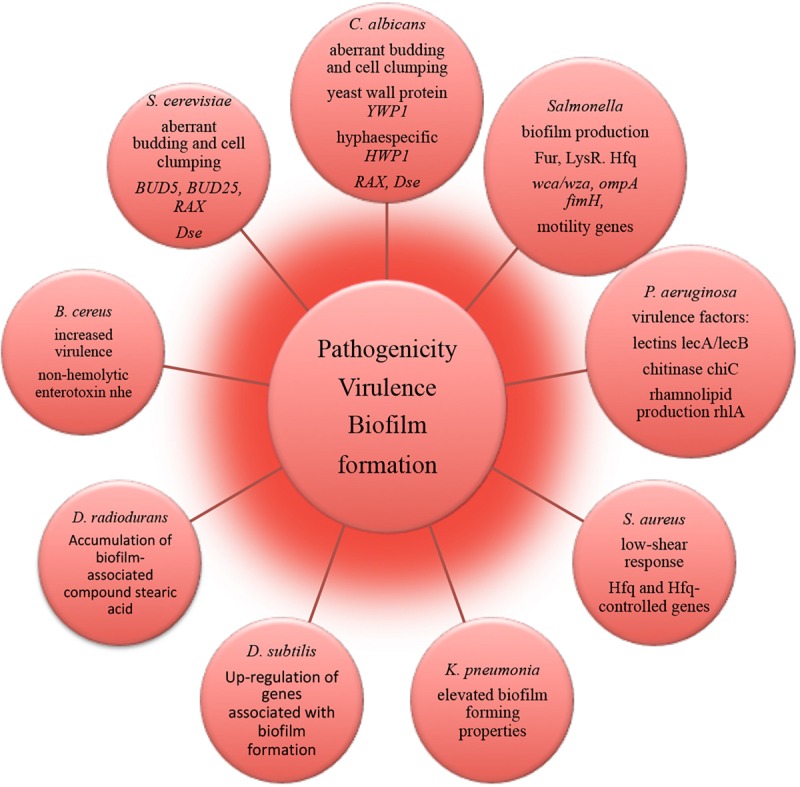
Molecular alterations underlying microbial pathogenicity, virulence and biofilm formation in outer space environment, resolved with –omics assisted investigations.

The molecular mechanisms underlying alterations of microbial virulence in space conditions have been successfully resolved with using state-of-the-art –omics technologies (Figure 4). A number of studies report that potentially pathogenic microorganisms display altered virulence and pathogenicity in real space or simulated microgravity conditions (Leys et al., 2004; Chopra et al., 2006; Taylor, 2015). Microbial morphogenic alterations have been described coherent with the enhanced pathogenicity of exposed microbes. Human opportunistic pathogen *Candida albicans* exhibited a morphogenic switch consistent with enhanced pathogenicity in simulated microgravity conditions (Altenburg et al., 2008). Microgravity induced the increased frequency of filamentous forms that contributes to the virulence and budding abnormalities (aberrant budding and cell clumping phenotype) of *C. albicans* and *S. cerevisiae* (Altenburg et al., 2008; Van Mulders et al., 2011). Spaceflight-cultured *C. albicans* exhibited random budding phenotype in accordance with the gene expression data (Crabbé et al., 2013). Genomic response analysis indicated that the expression of a set of genes involved in cell polarity and budding was significantly altered in these yeasts in simulated microgravity conditions. The conserved genes responsible for cell budding and separation were similarly down-regulated in microgravity exposed *C. albicans* and *S. cerevisiae*, thus suggesting that conservation of the genetic response takes place between the two distant yeast species (Altenburg et al., 2008; Van Mulders et al., 2011). Significant reduction in the expression of these genes appears to be consistent with the aberrant budding and cell-clumping phenotype of microgravity exposed cells. Increased virulence of *S. typhimurium* cultivated in spaceflight was accompanied by bacteria cellular aggregation, clumping and extracellular matrix formation, which is coherent with biofilm production (Wilson et al., 2007). Concomitantly, expression of *Salmonella* genes involved in biofilm formation was altered in microarray analysis during spaceflight (Wilson et al., 2007). A number of genes related to biofilm formation were up-regulated in transcriptomic profiling of *B. subtilis* during spaceflight aboard the ISS, which can be connected to the oxygen availability the in liquid cultures under the microgravity influence (Morrison et al., 2019).

The strain of human pathogen *K. pneumonia* returned from a spaceflight exhibited elevated biofilm forming properties as an important virulence characteristic (Li et al., 2014). The amount of biofilm-associated compound stearic acid (Saravanakumari and Mani, 2010; Vecino et al., 2015; Ge et al., 2016) was significantly elevated in cells of *D. radiodurans* exposed to vacuum and UVC-radiation (Ott et al., 2017). Although the cells of *D. radiodurans* do not naturally produce stearic acid in big quantities under non-stressed conditions (Melin et al., 1998), the observed accumulation of this biofilm-associated compound may lead to the high survival of *D. radiodurans* in dry multilayers under UVC/vacuum combined stress by preserving the structural integrity of cell membranes in conditions of vacuum-induced dehydration.

RNA-binding global regulatory protein Hfq has been shown to coordinate a regulatory response in bacterial reprogramming during spaceflight, altering bacterial gene expression and virulence under the influence of spaceflight conditions (multi-omics analyses in Wilson et al., 2007, 2008; Mastroleo et al., 2009; Crabbé et al., 2011). *P. aeruginosa* and human pathogen *B. cereus* cultured in the microgravity environment of spacecraft responded with the induction of several genes encoding virulence factors (Crabbé et al., 2011; Su et al., 2014). The obtained results of –omics studies of biofilm formation, microbial virulence and pathogenicity in space conditions and future follow-up investigations should continue to deliver novel molecular candidates for pharmacological intervention to prevent and control infectious diseases and to identify novel targets for vaccines and therapeutic development in order to keep crewmembers safe and healthy. This will ultimately facilitate long-term interplanetary transfer and productive space exploration.

## Space Systems Biology – a Framework for Integration of Outer Space Parameters With Omics Technology and Joined Mathematical Modeling

The –omics based approach has recently opened a window for a deep insight into molecular machinery implicated in survivability of space exposed microorganisms by revealing expression, metabolic functioning, and regulation of the genes and proteins encoded by the genomes of “space travelers.” Metabolic alterations mediated by genetic regulations affect the diverse biological activities of space-exposed microorganisms (Figure 1). Space induced metabolic rearrangements trigger the restoration of energy status of exposed microbial cell (Mastroleo et al., 2009; Wang et al., 2014; Zhang et al., 2015). Frequently, the exposed microbial entity is posed in “energy saving mode” by the regulatory molecular network in order to reduce the need for synthesis of cellular material in non-growing cells and complement the increased energy demands for the maintenance of genetic stability and cellular integrity in the space environment. Several global regulatory molecules have been identified which orchestrate the molecular response of few space-exposed microorganisms (Figure 2). Of especial attention is a group of hypothetical proteins of “unknown function” which are often numerically abundantly represented in space responses (over 50% of space exposed *B. subtilis* genes) (Nicholson et al., 2012). One of the strategies to assign novel functions for these hypothetical proteins might be the exploration of new space-related environmental and stress conditions.

Experimental parameters during space exposure affect microbial survival rates and may lead to certain discrepancies in –omics assisted analysis of returned/exposed microorganisms. The composition of cultivation medium influences the microbial space response (Benoit and Klaus, 2007; Baker and Leff, 2006; Wilson et al., 2008), for instance, by providing specific antioxidants presented in rich medium, which may protect microbial cell against ionizing radiation. The majority of space experiments have been performed on-board of spaceflights, where microorganisms are cultivated in protected environment of spacecraft (Table 1). Only a few microbial species were exposed unprotected to real space conditions outside the ISS and then subsequently investigated with –omics techniques (Nicholson et al., 2012; Vaishampayan et al., 2012). There is an increasing demand in new space experiments to broad our knowledge of molecular mechanisms of microbial survivability under the conditions of real outer space or its selected parameters. Concomitantly, the design of space exposure experiments has to be critically assessed to accommodate sufficient number of independent biological repetitions enabling a comprehensive statistical analysis of obtained –omics data. This is an extremely necessary prerequisite to avoid artifacts during the evaluation of the multitude of effects of outer space environment on microorganisms. In many instances, a multi-omics post-flight analysis faces the problem of limited amount of the microbiological samples exposed to the space environment. In this connection, the development of valid technical approaches enabling simultaneous efficient extraction of DNA, RNA, proteins, and metabolites from a minimal amount of microbial cells is highly desirable to overcome this limiting step (Weckwerth et al., 2004; Valledor et al., 2014; Ott et al., 2017). Another important issue, which requires critical reassessment, is the frequent absence of detailed reports on the environmental conditions during space exposure of microorganisms and corresponding ground control experiments. Due to the high sensitivity of the –omics techniques used in post-flight analysis and the partial occurrence of uncontrolled conditions of the space experiments, the appearance of stress-related artifacts cannot be ruled out. Providing of a record of controlled parameters (e.g., temperature, humidity, pressure profiles) during flight, simulated, and control experiments is highly anticipated to achieve a comprehensive and artifacts-free analysis of the effects of the space environment on physiology and molecular machinery of microorganisms.

**FIGURE 5:**
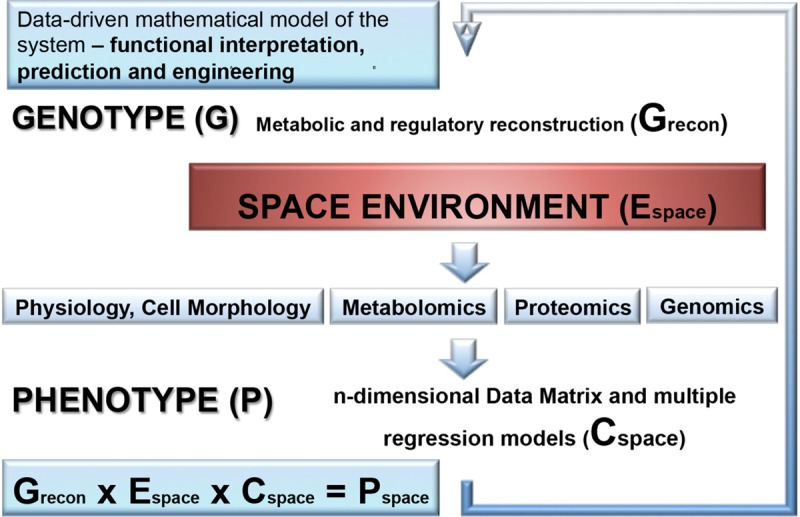
A combination of molecular data with a genome-scale metabolic reconstruction of the microbial species exposed to the space environment. First, genome-scale metabolic and regulatory reconstruction of the respective microorganism is performed based on the available genome sequence and gene annotation information and results into a genotype matrix (G_recon_). This provides the basic predictive metabolic model to eventually integrate statistical models of molecular data generated by OMICS technology, physiological and morphological data as well as environmental parameter which are accurately monitored in outer space conditions. The molecular and physio-morphometric data are correlated with environmental parameter by multiple regression methods and generate a comprehensive data covariance matrix times environmental data matrix (C_space_ × E_space_). The resulting data covariance model is eventually connected with the genotype matrix G_recon_ to generate a biomathematical data-driven regulatory and predictive model for the response trajectory of the microorganism in outer space conditions (P_space_). Overall, this is an iterative process improving step by step the predictive models (for further information see Weckwerth et al., 2004; Weckwerth and Morgenthal, 2005; Weckwerth, 2011a, 2016, 2019; Sun and Weckwerth, 2012; Doerfler et al., 2013; Nägele et al., 2014, 2016; Sun et al., 2015).

In addition to the proteotranscriptomic profiling, we propose that new space experiments should include a detailed metabolomic analysis of exposed microorganisms. This novel approach provides a rich source of new findings of fine molecular network regulating the space response (Ott et al., 2017, 2019a,b). Another aspect which is not yet addressed is the combination of molecular data with a genome-scale metabolic reconstruction of the respective species which is rather routine standard nowadays for the analysis of organisms (Weckwerth, 2011b). Summarizing the comprehensive review of metabolic alterations of microorganisms in space conditions reveals a multifactorial response trajectory which is not intuitively leading to a causal understanding. Thus, it is of utmost importance to integrate isolated biochemical parts into a global statistical and mathematical model. Here, we propose hybrid modeling approaches integrating statistical regression models and genome-scale metabolic reconstruction (Weckwerth, 2019). In Figure 5 the proposed workflow for this hybrid modeling approach is shown: (i) for microorganisms with the available genome sequences genome-scale metabolic reconstruction is a straightforward approach (Thiele and Palsson, 2010; Weckwerth, 2011b); (ii) in a next step, –omics technology is applied as well as classical physiological and morphological parameter are measured to build a comprehensive statistical regression model of the response trajectory to environmental space conditions. Decisive for this regression model is the experimental design and the comprehensive monitoring of the environmental parameters. (iii) Finally, the statistical and the genome-scale models are joined by biomathematical approaches to represent a global model of metabolic regulation in outer space conditions (Weckwerth, 2019; Wilson et al., 2020). Based on a thorough hybrid modeling approach of the microbial systems in outer space as described above also metabolic engineering strategies can be developed upon revealing the elements of adaptation: from intrinsic protective mechanisms to elevated repair abilities, and a merging of these adaptation strategies.

## Author Contributions

Both authors conceived the review, conducted the literature search, created the figures, wrote and revised the final version of the manuscript.

## Conflict of Interest

The authors declare that the research was conducted in the absence of any commercial or financial relationships that could be construed as a potential conflict of interest.
